# Advances in Soft Tissue and Bone Sarcoma

**DOI:** 10.3390/cancers16162875

**Published:** 2024-08-19

**Authors:** Catrin S. Rutland

**Affiliations:** 1 School of Veterinary Medicine and Science, Faculty of Medicine and Health Sciences, University of Nottingham, Nottingham NG7 2RD, UK; catrin.rutland@nottingham.ac.uk; 2 Faculty of Medicine and Health Science, Biodiscovery Institute, University of Nottingham, Nottingham NG7 2RD, UK

## 1. Introduction

This *Cancers* Special Issue on bone and soft tissue sarcomas highlights the latest discoveries in soft tissue and bone cancers from the laboratory through to the clinics, from bench to bedside, and beyond. Despite advances in clinical and basic research, soft tissue and bone sarcomas remain clinically challenging. This Special Issue brings together original research and reviews covering bone and soft tissue sarcomas on topics ranging from detection and diagnosis methods using histopathology, imaging, and molecular advances through to treatment and prognosis techniques, including treatment efficacy and survival outcome analysis.

Both primary bone cancers and soft tissue sarcomas are relatively rare, each accounting for less than 1% of malignancies [1]. Not only are sarcomas relatively rare, but they have differing locations in the body and different characteristics and subtypes; more than 100 subtypes of soft tissue sarcomas have been classified to date [2,3]. Sarcomas are therefore often difficult to diagnose, characterise and treat.

The research being conducted is as diverse as the sarcomas themselves. Patient samples and datasets, both in vivo and in vitro, models including organoids and organ chips [4,5,6,7,8], mathematical and bioinformatics models [9,10], and clinical trials along with cohort studies are being used alongside machine-aided learning, including in areas such as radiomics, biomarkers and next-generation sequencing-based methods [11,12,13,14,15]. Advances in imaging techniques such as surgery, magnetic resonance imaging (MRI) and positron emission tomography (PET), and molecular imaging technology such as PET tracers [8,16,17], are also improving diagnostic, prognostic, surgical and drug development tools and approaches. Advances are being achieved in drug discovery and personalised medicine, including in targeted therapies, immunotherapy, chimeric antigen receptor (CAR) T-cell therapy, tumour-infiltrating lymphocytes (TILs), vaccines and combination therapies, to name just a few [10,17,18], and developments and innovations in genetic testing, molecular profiling and epigenetic aspects [3,19] of sarcomas are needed. A deeper understanding of mechanisms of resistance and research into differing sub-types and the tumour microenvironment is also essential to move sarcoma research and clinical approaches forward. More novel approaches to diagnostics, prognostics and therapeutics are essential looking forward, as scientific discoveries are translated into the clinic.

This “Bone and Soft Tissue Sarcomas” *Cancers* Special Issue (https://www.mdpi.com/journal/cancers/special_issues/C8D9NZ20HW accessed on 15 August 2024) consists of 14 papers with article submissions accepted until 30th April 2024. It explores the advances in diagnosis, prognosis, mechanisms and treatment outcomes for bone and soft tissue sarcomas. The research covers bone sarcomas such as chordomas, which are rare malignant neoplasms, through to the more common osteosarcoma. This Special Issue also covers soft tissue sarcomas, such as one of the most common sub-types of soft tissue sarcoma—undifferentiated pleomorphic sarcoma—through to rare sarcomas within the peritoneal cavity.

## 2. An Overview of the Articles Published in This Special Issue

The key themes of this special issue are classification, diagnostic and prognostic advances and indicators (Contributions 1, 2, 3, 5, 9, 10, 12, 13 and 14), key areas of therapeutic development (6, 7, 8, 12 and 14) and treatment performance and outcomes (contributions 4, 7, 8 and 11). Contributions 1–6 represent a systematic review and reviews of the field, whereas Contributions 7–14 are original research articles focusing on in vitro and in vivo models or patient cohorts.

Contribution 1 in this Special Issue is ‘Soft Tissue Sarcoma Mimicking Melanoma: A Systematic Review’ by Cassalia and coauthors. Contribution 2, ‘Predictors of Symptomatic Venous Thromboembolism in Patients with Soft Tissue Sarcoma in the Lower Extremity’, is a review by Kamalapathy and coauthors. Kim et al., present ‘Classification of Chondrosarcoma: From Characteristic to Challenging Imaging Findings’ in Contribution 3, while Contribution 4 outlines the ‘Current Landscape of Immunotherapy for Advanced Sarcoma’ as reviewed by Albarrán and colleagues. Contribution 5, by Costci et al., is ‘Gender Differences in Soft Tissue and Bone Sarcoma: A Narrative Review’ and the final review, Contribution 6 by Lesovaya and coauthors, is ‘Genetic, Epigenetic and Transcriptome Alterations in Liposarcoma for Target Therapy Selection’.

Contribution 7 is a research article on ‘Treatment Pathways and Prognosis in Advanced Sarcoma with Peritoneal Sarcomatosis’ by Klingler and colleagues. Sarcomas within the peritoneal cavity are not only rare but remain difficult to treat due to their differing subtypes and characteristics. This research, focusing on surgical procedures, presents 19 patients with peritoneal sarcomatosis, outlining their journeys from diagnosis and treatment through to outcomes, in order to share management practices with others.

Contribution 8, by Polera et al., also looks at potential treatment pathways in the paper ‘The First-In-Class Anti-AXL×CD3ε Pronectin™-Based Bispecific T-Cell Engager Is Active in Preclinical Models of Human Soft Tissue and Bone Sarcomas’. This article explores AXL, a TAM family tyrosine kinase receptor, as a target for an innovative immunotherapeutic strategy. These in vitro and murine in vivo studies have indicated the antitumor efficacy of pAXL×CD3ε against sarcoma cells, which potentially represents a new-generation strategy for managing sarcomas.

Brookes and coauthors explored ‘What Is the Significance of Indeterminate Pulmonary Nodules in High-Grade Soft Tissue Sarcomas? A Retrospective Cohort Study’ in Contribution 9. Understanding the roles of indeterminate pulmonary nodules in high-grade soft tissue sarcoma, whether they may be benign or malignant, may impact clinical decision making. The important conclusions of this research, looking at 389 patients, indicate that in patients with grade 3 soft tissue sarcomas, significantly worse overall survival was observed, as was an increased risk of developing lung metastases. These significant differences were not observed in grade 2 patients presenting with indeterminate pulmonary nodules. Clinically, the authors indicated that the primary tumour must be considered alongside indeterminate pulmonary nodules when considering risk progression, and determined that monitoring via CT scans at 6 and 12 months would be advisable.

Contribution 10, by Iiuz et al., investigated ’Rapid Classification of Sarcomas Using Methylation Fingerprint: A Pilot Study’. This research article explored the potential for methylation and copy-number variation data in terms of rapid point-of-care sarcoma classification. The end goal was to reduce the time taken to classify sarcomas in order to commence appropriate treatments in a more time efficient manner, and potentially to expand the tools available for classification.

Contribution 11, ‘High-Grade Pleomorphic Sarcomas Treated with Immune Checkpoint Blockade: The MD Anderson Cancer Center Experience’, by Nasr and coauthors, investigated one of the most common soft tissue sarcomas. Their work included 26 undifferentiated pleomorphic sarcoma patients and 10 patients with other high-grade pleomorphic sarcomas. This retrospective study indicated that immune checkpoint blockade (ICB) treatment resulted in significantly improved progression-free survival. Toxicity was manageable, with no patient deaths. Notably, their data also indicated that compared to previous clinical trials, their data showed that a combination treatment seemed inferior to standalone ICB regarding progression-free survival.

Contribution 12, by de Brot and colleagues, investigated three potential diagnostic and prognostic indicators, highlighting prospective therapeutic targets in their article ‘Immunohistochemical Investigation into Protein Expression Patterns of FOXO4, IRF8 and LEF1 in Canine Osteosarcoma’. Using immunohistochemistry alongside quantitative H-scoring and qualitative analysis, their research showed the expression of FOXO4, IRF8 and LEF1 in osteosarcoma. Their work highlighted IRF8 and LEF1 as particularly promising biomarker candidates and therapeutic targets, given their expression patterns, mechanisms and involvement in a number of molecular pathways.

Contribution 13 by Yoon et al. researched 65 patients in ‘Quantitative Bone SPECT/CT of Central Cartilaginous Bone Tumors: Relationship between SUVmax and Radiodensity in Hounsfield Unit’. This contribution focused on accurately grading cartilaginous bone tumors using SPECT/CT. Their research revealed a negative correlation between SUVmax and radiodensity in HU measurements in central cartilaginous bone tumours, and patients with a higher SUVmax and lower HUSD were more likely to have a malignant cartilaginous bone tumour. This highlighted the diagnostic and prognostic uses of this technique in central cartilaginous bone tumours.

The final article published, Contribution 14, was ‘Conventional spinal chordomas: investigation of SMARCB1/INI1 protein expression, genetic alterations in SMARCB1 gene and clinicopathological features in 89 patients’ by Maioli and coauthors. This research determines the immunohistochemical expression of SMARCB1/INI1 and fluorescence in situ hybridisation (FISH) to understand the underlying genetic alterations in the SMARCB1 gene in 89 patients with conventional spinal chordomas. This supports the information relating to the potential of SMARCB1/INI1 as a target for molecular therapy.

## 3. Conclusions

Many of the studies published in this Special Issue made advances and recommendations relating to diagnosis, prognosis and therapeutics. Directions for future research included the need for research into differing subtypes of sarcomas and for more varied cohorts in general. Work completed in vitro and in vivo has highlighted the need for the development of promising tools and for more translational research to be conducted (such as Contributions 8 and 12). Furthermore, research such as that presented in Contribution 11 has demonstrated the need for cohort studies following clinical trials. Contribution 7 highlights the need for large soft tissue sarcoma databases, especially in relation to future research on subtypes and progression, to support evidence-based approaches for management and tailored treatment plans. The papers in this *Cancers* Special Issue also highlight the complexities faced in classifying and characterising sarcomas, which in turn complicate the discovery of molecular mechanisms and pathways, which makes finding biomarkers more difficult, and additionally makes it challenging to discover and develop effective treatment options.

Generalised 5-year survival rates for soft tissue sarcomas (2000–2018) ranged from 82% for localised to 59.6% for regionalised and 16.7% for distant sarcomas; for bone and joint, these survival rates ranged from 82.6% for localised to 67.4 for regional and 30.8% for distant [20]. With these survival rates in mind, there is still much research needed into the diagnosis, prognosis and treatment tools required to enhance healthcare options.
